# Environmental and Health Risk Assessments of Volatile Organic Compounds (VOCs) Based on Source Apportionment—A Case Study in Harbin, a Megacity in Northeastern China

**DOI:** 10.3390/toxics14010046

**Published:** 2025-12-31

**Authors:** Jinpan Jiang, Bo Li, Binyuan Wang, Lu Lu, Fan Meng, Chongguo Tian, Hong Qi, Ai-Ling Lian

**Affiliations:** 1 School of Environment, Harbin Institute of Technology, Harbin 150090, China; 2 State Key Laboratory of Urban-Rural Water Resource and Environment, Harbin Institute of Technology, Harbin 150090, China; 3 School of Environment, Tsinghua University, Beijing 100084, China; 4 Key Laboratory of Coastal Environmental Processes and Ecological Remediation, Yantai Institute of Coastal Zone Research, Chinese Academy of Sciences, Yantai 264003, China; 5 Department of Operating Room, First Affiliated Hospital of Harbin Medical University, Harbin 150001, China

**Keywords:** VOC characterization, source apportionment, environmental and health risk assessment, mitigation policy

## Abstract

The multiple sources and concomitant negative environmental and health impacts of volatile organic compounds (VOCs) in the atmosphere demonstrate their importance in air pollution control. This study employed environment- and health risk-oriented source apportionment methods to quantitatively estimate VOCs’ contribution to air pollution and health risks, using offline VOC measurements from the Harbin urban region from 2021 to 2022. Total volatile organic compounds (TVOCs) averaged 25.6 ± 8.2 ppb, except for alkanes (34.4%), and aromatics (24.2%) were found to be a major contributor, with the highest L_OH_ (38.0%), ozone formation potential (OFP) (43.0%), and secondary organic aerosol formation potential (SOAFP) (95.0%) and exerting a directly toxic effect (46.0%). Positive matrix factorization (PMF) source apportionment revealed that vehicle exhausts, combustion sources, solvent and coating usage, solvent and fuel evaporation, and petrochemical industry sources were key VOC sources. A health risk assessment showed that there was an integrated carcinogenic risk of 5.8 × 10^−4^, with respiratory (1.5 × 10^−4^) and hematologic systems (1.5 × 10^−4^) representing higher carcinogenic risks. Both benzene and naphthalene exhibited carcinogenic risks of 1.5 × 10^−4^, implying an excess of higher cancer risk levels (1.0 × 10^−4^). Significant joint environmental and health benefits could be obtained by reducing benzene and naphthalene concentrations by about 50.0%, along with the abatement of vehicle exhausts (82.6%), combustion sources (40.7%), and solvent and coating usage (50.7%). This study can serve as useful guidance for the quantitative mitigation of hazardous VOCs and their key sources.

## 1. Introduction

The atmospheric composite pollution system, formed by fine particulate matter (PM_2.5_) and ozone (O_3_) through multi-scale coupling interactions with a meteorological climate system, has limited air quality improvements in China. Due to their “homologous origin”, they share volatile organic compounds (VOCs) as key precursors. On the one hand, active VOCs are oxidized by NO_x_ and radicals, disturbing the NO–NO_2_–O_3_ equilibrium and ultimately generating O_3_ [1,2], which exerts multiple negative effects on agricultural production and ecosystems by strengthening atmospheric oxidation [3,4]. On the other hand, low/semi-volatile organic compounds (LVOCs/SVOCs), derived from primary VOCs via the oxidation pathway, can be transformed into secondary organic aerosols (SOAs), the main organic component of PM_2.5_, through multiphase processes [5,6,7], which could accelerate haze formation and significantly impact climate change through the radiative forcing effect [8,9,10]. Moreover, epidemic studies have confirmed that O_3_, PM_2.5_, and VOCs cause health issues such as hematologic, respiratory, and nervous diseases through multi-pathway toxic effects [11,12,13]. Therefore, reducing VOC emissions is essential for synergistic PM_2.5_-O_3_ pollution control and to protect human health.

VOCs in the atmosphere exhibit multi-source emission characteristics, driven by both natural and anthropogenic sources. With the rapid development of urbanization, anthropogenic emissions play a dominant role in urban and surrounding areas, and highly varied sources are present in different regions [14]. Recently, source apportionment studies [15] have found that vehicle emissions dominate in northwestern (35.1 ± 32.9%) and central (24.9 ± 8.2%) China, while eastern (22.6 ± 11.4%) and southern (26.3 ± 3.2%) China have been more impacted by industrial emissions or petrochemical industrial sources. The most important sources in northern and southwestern China are combustion sources (30.0 ± 10.3%) and LPG/NG sources (34.0 ± 2.7%). Nevertheless, studies in China have focused on more economically developed areas, such as the Beijing–Tianjin–Hebei, Yangtze River Delta, and Pearl River Delta regions [16,17,18]. There is still a relative lack of research on VOC source apportionment in the atmosphere in extremely cold cities in northeastern China.

In addition, different VOC compositions, such as OVOCs and aromatics, actively participate in chemical reactions related to secondary formation [19], which significantly contribute to the formation of photochemical smog. Most previous studies have assessed the environmental impacts of VOCs from one or two perspectives. For instance, Bi et al. [20] only employed ozone formation potential (OFP) to describe secondary pollution from VOCs in Nanjing, China; however, environmental effects, such as L_OH_, secondary organic aerosol formation potential (SOAFP), and direct toxicity, remain unclear due to being largely incomplete. In this regard, a multi-effect assessment system, including L_OH_, OFP, SOAFP, and toxic effects [21], should be adopted to comprehensively characterize the integrated environmental impact of VOCs.

Most importantly, each VOC emission source has a unique combination of VOC species with different environmental and organ-specific health impacts [22]. Despite extensive studies on the environmental and health-related risks of VOCs [23,24], it is worth noting that past works have rarely included target-organ-specific health risk assessments and have only qualitatively proposed VOC control strategies based on source apportionment or health risk assessment results, which might make mitigation policies less efficient. Consequently, the target-organ-specific health risks of VOCs should be further investigated using quantitative assessments of the health risks and environmental impact of each VOC source to assist policy makers in optimizing abatement strategies.

To address these research gaps, we conducted offline monitoring of VOCs in the urban atmosphere of Harbin in 2021–2022. Our aim was to (1) determine the VOC concentration in Harbin; (2) conduct environmental and health risk assessments of VOCs; and (3) identify sources of VOCs and determine which VOC species/sources contribute the most to environmental and health risks. The findings of this study, obtained based on quantitative evidence from environmental and health risk assessments, are useful for improving VOC pollution management policies in Harbin.

## 2. Materials and Methods

### 2.1. Study Site Description

As a representative high-latitude city in China, Harbin features a temperate monsoon climate. It has a long and frigid winter, while its summer is short and cool. The autumn, a transitional season, is characterized by sharp temperature fluctuations. With a maximum annual temperature difference of up to 60 °C, Harbin is recognized as a typical cold city in China. To investigate the concentration, sources, and environmental and health effects of VOCs in the atmosphere of Harbin, the sampling site is located on the roof of the School of Environment, Harbin Institute of Technology (126.69° E, 45.76° N), approximately 30 m above sea level and about 50 m away from the off-campus road (Supplemental Figure S1). It is located in a mixture of transportation, residential, and commercial areas, without any large emission sources such as chemical or thermal power plants nearby. Additionally, the site has a broad field of view and favorable diffusion conditions, making it a good representation of VOC pollution levels in the urban atmosphere of Harbin.

### 2.2. VOC Sampling and Analysis

To determine the temporal characteristics of the VOC concentration in Harbin, offline monitoring was conducted from April 2021 to January 2022. The sampling campaign was conducted during both the non-heating and heating period. The non-heating period lasted from 18 April to 17 October 2021, while the heating period was from 3 November 2021 to 23 January 2022. During the sampling period, samples were collected on Wednesdays and Sundays every week to represent weekdays and weekends, respectively. In order to explore daily variation trends, samples were collected at 8:30 AM, 1:30 PM, and 18:30 PM on the sampling day. A total of 108 samples were collected during the non-heating period, and 66 samples were collected during the heating period, making a total of 174 samples throughout the sampling period (Supplemental Table S1).

The air samples were collected instantaneously using cleaned 6 L summa canisters (Restek, Bellefonte, PA USA). After sampling, CO_2_, CO, H_2_O, O_2_, and N_2_ were removed with a cryogenic pre-concentrator (UNITY-xr, MARKES, Bridgend UK), and the volume of the VOCs was further concentrated. They were then analyzed by gas chromatography–mass spectrometry (GC-MS, Agilent 6890N-5975B, Santa Clara, CA USA). In total, 94 target VOCs were quantified in this study: 26 alkanes, 26 halocarbons, 24 aromatics, 10 OVOCs, 7 alkenes, and 1 sulfide. Standard curves were constructed for target VOCs using a photochemical assessment monitoring station (PAMS) and U.S. EPA TO-15 standard substances, with R^2^ ≥ 0.99 for all compounds. Quality assurance and quality control, including summa canister cleaning, laboratory and transportation blanks, and parallel sample determination, were performed according to HJ 759-2015 [25]. The MDLs (0.002 ppb–0.038 ppb) and RSDs (2–7%) of target VOCs are shown in Table S2. During the sampling periods, meteorological parameters and air pollutant concentrations were obtained from the National Meteorological Science Data Center (http://data.cma.cn/, last access: 12 November 2025) and the China National Environmental Monitoring Center (https://www.cnemc.cn/, last access: 12 November 2025).

### 2.3. Multi-Effect Assessment of Environmental Risks of VOCs

The rate of hydroxyl radical loss (L_OH_) in VOCs can be calculated using Equation (1):

(1) $$L_{\mathrm{OH}}\;=\;\sum_{i}^{n}k_{{\mathrm{OH}}_{i}}\;\times \;{\left[\mathrm{VOC}\right]}_{i}$$

where $k_{{\mathrm{OH}}_{i}}$ is the rate constant [26,27] for the reaction of VOC_i_ with ·OH, [VOC]_i_ is the concentration of the ith VOC species, and n is the number of VOCs.

The ozone formation potential (OFP) of VOCs was used to calculate the contribution to O_3_ production based on Equation (2):

(2) $$\mathrm{OFP}=\sum_{i}^{n}{\mathrm{MIR}}_{i}\;\times \;{\left[\mathrm{VOC}\right]}_{i}$$

where MIR_i_ is the maximum incremental reactivity [28] of VOC_i_.

The secondary organic aerosol formation potential (SOAFP) of VOCs can be calculated as shown in Equation (3):

(3) $${\mathrm{SOAFP}}_{i}\;=\;\frac{\mathrm{increment}\;\mathrm{in}\;\mathrm{SOA}\;\mathrm{mass}\;\mathrm{concentration}\;\mathrm{with}\;\mathrm{species}\;i}{\mathrm{increment}\;\mathrm{in}\;\mathrm{SOA}\;\mathrm{with}\;\mathrm{toluene}}\;\times \;100$$

where SOAFP_i_ is the potential for VOC_i_ to yield SOA relative to the equivalent mass of toluene [27,29,30].

The toxicity of VOC species is categorized into four grades based on information from European Commission and International Agency for Research on Cancer (IARC) about the carcinogenic, teratogenic, and mutagenic properties of different VOCs. Grade 1 represents IARC group 3; grade 2 corresponds to IARC group 2B (possibly human carcinogens); grade 3 belongs to IARC group 2A (likely human carcinogens); and grade 4 represents IARC group 1 carcinogens (highly toxic to humans) [21,31]. The relative toxic effect can be calculated using Equation (4):

(4) relative toxicity effect = [VOCs] × toxicity grade

Calculation parameters relating to the assessment of the integrated environmental effects of VOCs are detailed in Table S3.

### 2.4. Health Risk-Oriented Source Apportionment

Previous studies have only qualitatively suggested VOC control measures based on either source apportionment or health risk assessment results, with very few quantitative and source-specific mitigation strategies being proposed. Therefore, we employed a new health risk-oriented source analysis method—combining receptor modeling with risk assessment—to quantify the source-specific health risks of hazardous VOCs [22,32].

#### 2.4.1. Source Apportionment

This study used a cross-validation source identification method based on positive matrix factorization (PMF), using cross-validation factor contributions with characterized species ratios and reference emission hotspots of local industry and social activities, to minimize the uncertainties associated with the arbitrary interpretation of PMF factors. The model is described in the EPA PMF 5.0 user guide [33], and summaries of the PMF error estimation diagnostics using our VOC dataset are shown in Tables S5–S7.

#### 2.4.2. Health Risk Assessment

Hazard quotients (HQs) and inhalation cancer risks (ICRs) were calculated using epidemiological models [34].

(5) $${\mathrm{HQ}}_{i}=\frac{C_{{\mathrm{VOC}}_{i}}}{{\mathrm{REL}}_{i}}$$

where $C_{{\mathrm{VOC}}_{i}}$ and ${\mathrm{REL}}_{i}$ are concentrations and reference exposure levels of the ith VOC species (µg/m^3^).

(6) $$\mathrm{ICR}={\;\mathrm{DOSE}}_{\mathrm{air}}\;\times \;\mathrm{CPF}\;\times \;\mathrm{ASF}\;\times \;\frac{\mathrm{ED}}{\mathrm{AT}}\;\times \;\mathrm{FAH}$$

(7) $${\mathrm{DOSE}}_{\mathrm{air}}={\;C}_{{\mathrm{VOC}}_{i}}\;\times \;\frac{\mathrm{BR}}{\mathrm{BW}}\;\times \;A\;\times \;\mathrm{EF}\;\times {\;10}^{-6}$$

where DOSE_air_ represents the dose through inhalation (mg/kg/d), expressed as the multiplication of $C_{{\mathrm{VOC}}_{i}}$, $\frac{\mathrm{BR}}{\mathrm{BW}}$ (breathing rate normalized to body weight; L/kg body weight-day), A (inhalation absorption factors; unitless), and EF (exposure frequency; unitless). CPF is the inhalation cancer potency factor (mg/kg-day^−1^), ASF represents the age sensitivity factor (unitless), and ED, AT, and FAH represent the exposure duration (years), average time for cancer risk (years), and fraction of time spent at home (unitless), respectively.

The parameters relating to our health risk calculation are detailed in Tables S8 and S9.

#### 2.4.3. Preliminary Remediation Goals

Preliminary remediation goals (PRGs), also known as risk-based concentrations, represent the maximum concentrations of toxics that are allowed to be left at a given site without threatening public health [35,36].

(8) $$\mathrm{PRGs}=\frac{{\mathrm{ICR}}_{\mathrm{target}}\;\times \;\mathrm{AT}}{\left(\mathrm{BR}/\mathrm{BW}\right)\;\times \;A\;\times \;\mathrm{EF}\;\times \;\mathrm{CPF}\;\times \;\mathrm{ASF}\;\times \;\mathrm{ED}\;\times \;\mathrm{FAH}\;\times \;10^{-6}}$$

where ICR_target_ refers to WS/T 777-2021 [37], with 1.0 × 10^−6^, 1.0 × 10^−5^, and 1.0 × 10^−4^ representing lower, moderate, and higher cancer risks, respectively.

Based on the difference between PRGs and observed concentrations, the hazardous VOCs or source reduction rates for cancer risks exceeding those defined in WS/T 777-2021 were calculated using Equation (9):

(9) $$\frac{\mathrm{PRGs}-\mathrm{Obs}}{\mathrm{Obs}}\;\times \;100\%$$

## 3. Results

### 3.1. Characterization of VOCs

#### 3.1.1. Characterization of VOC Compositions and Hazardous VOC Levels

**Figure 1:**
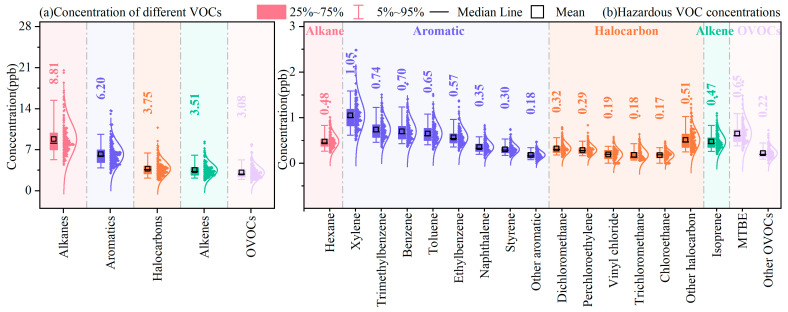
Concentrations of (**a**) major VOC chemical groups and (**b**) hazardous VOC species during the observation period.

The concentrations of different VOC groups are presented in Figure 1a. TVOCs ranged from 12.6 to 59.1 ppb, with the average being 25.6 ± 8.2 ppb, while alkanes reached 8.8 ± 2.9 ppb (34.4%); n-butane, iso-butane, n-pentane, and iso-pentane contributed significantly, which could be due to their widespread emission sources and long atmospheric lifetimes. The second-largest group was aromatics (6.2 ± 1.9 ppb, 24.2%), whose dominant compounds were benzene, toluene, ethylbenzene, xylene, and naphthalene, possibly originating from vehicle exhausts and combustion sources. Halocarbons (3.8 ± 1.3 ppb) contributed 14.7% to TVOCs, with chloromethanes (CH_3_Cl, CH_2_Cl_2_, CHCl_3_, and CCl_4_) being the major compounds. Together, alkenes (3.5 ± 1.2 ppb) and OVOCs (3.1 ± 1.1 ppb) accounted for 25.7%, with 1-pentene, trans-2-butene, isoprene, acetone, and MTBE being the key species.

Furthermore, the time series of meteorological parameters and air pollutant concentrations during the sampling period are shown in Figure S2. The ambient temperature ranged from −25.9 to 31.1 °C, the RH varied between 21% and 100%, and prevailing winds shifted between southeasterly and southwesterly, with a WS of 0–7.9 m/s. O_3_ was 28–289 µg/m^3^, with 3 days exceeding the ambient air quality standards’ [38] secondary limit (160 µg/m^3^): 21 April (231.33 µg/m^3^), 19 May (164.33 µg/m^3^), and 23 May (229 µg/m^3^). PM_2.5_ ranged from 5 to 252 µg/m^3^, with 6 days above the national limit (75 µg/m^3^) [38], mainly in April, November, and December 2021 and January 2022. Among them, PM_2.5_ was 231.67 µg/m^3^ and PM_10_ was 274 µg/m^3^ on 21 April. Considering local production activities and meteorological parameters, there was significant straw burning around Harbin on 21 April, with a suitable temperature (about 23 °C), sufficient sunlight, and light wind (1.5–1.8 m/s), which aggravated the generation and accumulation of O_3_ and PM near the surface. Particularly, CO (a typical combustion tracer [39]) concentrations reached up to 1700 µg/m^3^, also indicating that straw burning emissions make significant contributions to O_3_ and PM exceedances.

In Figure S3, we show that VOCs positively correlate with PM (*R_spearman_* for PM_2.5_ was 0.64, *p* ≤ 0.001; *R_spearman_* for PM_10_ was 0.41, *p* ≤ 0.001). It was well documented that greater VOC concentrations signify increasing rates of PM production via photochemical oxidation, gas-particle partitioning, and/or non-homogeneous absorption [40,41]. The positive correlations of VOCs with SO_2_ (*R_spearman_* = 0.68, *p* ≤ 0.001) and CO (*R_spearman_* = 0.51, *p* ≤ 0.001) indicate potential co-sources such as vehicle exhausts and combustion sources [42,43]. While VOCs correlated negatively with O_3_ (*R_spearman_* = −0.60, *p* ≤ 0.001) and temperature (*R_spearman_* = −0.83, *p* ≤ 0.001), O_3_ correlated positively with temperature (*R_spearman_* = 0.64, *p* ≤ 0.001), suggesting that secondary transformation of VOCs to O_3_ was more likely under favorable conditions (e.g., higher temperatures, higher VOC concentrations) [44]. Moreover, VOC/NO_x_ values were almost all below 10, demonstrating that O_3_ formation was sensitive to VOCs [45]; therefore, reducing VOC emissions would be beneficial for O_3_ mitigation.

**Figure 2:**
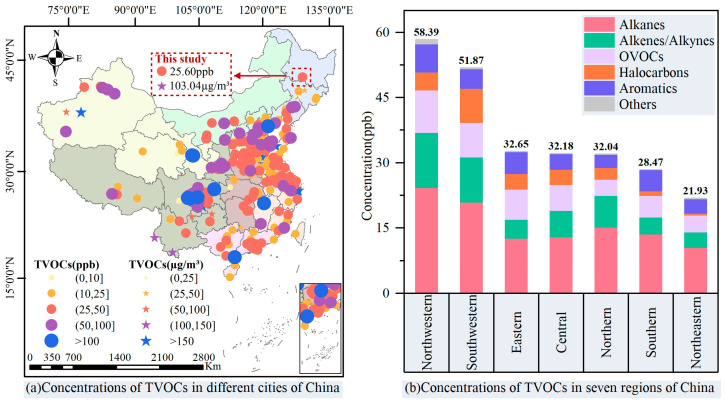
Distribution of TVOC levels in China, summarized from the literature.

In order to compare the differences in VOC concentrations in Harbin with other regions of China, we summarized the TVOC concentrations of seven regions of China, as shown in Figure 2, through searches of Web of Science, China National Knowledge Infrastructure (CNKI), and other databases (for more details, see “SI—Literature data—Figure 2”).

Within the scope of our literature research, TVOC levels in China ranged from 6.4 to 146.5 ppb. The northwestern and southwestern regions had the highest TVOC levels, with 58.4 ± 27.1 ppb and 51.9 ± 30.5 ppb, respectively, while the northeastern regions had the lowest levels with 21.9 ± 14.2 ppb, which was less than half of those in the northwestern or southwestern regions. As noted above, VOC pollution (25.6 ± 8.2 ppb) in the atmosphere of Harbin was at the lower-middle level in China and much lower than those of the Guanzhong Plain, Yangtze River Delta, Pearl River Delta, and Sichuan Basin regions. Within northeastern China, TVOC levels were higher than in Dalian [46] (TVOCs: 16.4 ppb) and Changchun [47] (TVOCs: 14.5 ppb) but significantly lower than in Shenyang [48] (TVOCs: 50.2 ppb), suggesting that as a representative of megacities in northeastern China, VOC pollution in Harbin should be given significant attention.

While TVOCs provide a snapshot of the total concentrations of all detectable VOCs, the levels of hazardous species are more relevant when determining the health effects of VOCs. Therefore, with consideration of the International Agency for Research on Cancer (IARC)’s classification, we screened 32 hazardous VOCs, including n-hexane, xylene (o-xylene, m-xylene, p-xylene), trimethylbenzene (1,3,5-trimethylbenzene, 1,2,4-trimethylbenzene, 1,2,3-trimethylbenzene), benzene, toluene, ethylbenzene, naphthalene, styrene, dichloromethane, perchloroethylene, vinyl chloride, trichloromethane, chloroethane, isoprene, and MTBE, as shown in Figure 1b.

The total concentration of thirty-two hazardous VOCs was 8.0 ± 2.5 ppb, accounting for 31.3% of TVOCs. Given that the sampling site was downtown, it can be inferred that anthropogenic emissions have a significant impact on local air quality and may pose a significant risk to human health. Among the thirty-two hazardous VOCs, xylene was the most abundant, accounting for 13.1% of the total hazardous VOCs, followed by trimethylbenzene (9.2%), benzene (8.8%), toluene (8.2%), MTBE (8.1%), ethylbenzene (7.1%), n-hexane (6.0%), isoprene (6.0%), naphthalene (4.4%), dichloromethane (4.0%), and perchloroethylene (3.6%).

The high levels of aromatic groups, including xylene, trimethylbenzene, benzene, toluene, and ethylbenzene, were expected, especially in large urban areas, owing to vehicle exhausts and widespread usage of volatile chemical products (VCPs) such as cleaning agents, solvents, adhesives, and coatings [30]. Furthermore, n-hexane is mainly associated with conventional oil and gas production and refinery emissions [49]. MTBE is extensively used as gasoline additive [50], dichloromethane is widely applied for industrial solvents [51], and perchloroethylene is commonly employed as a dry cleaning solvent [52]. Meanwhile, naphthalene and isoprene are associated with biomass combustion emissions [53]. Consequently, anthropogenic emissions from VCP usage, traffic, industry, and biomass combustion increase the ambient levels of hazardous VOCs, resulting in an increased exposure risk.

#### 3.1.2. Temporal Variations in VOCs

Being representative of an extremely cold city, heating is used in Harbin for up to about 6 months a year; the sampling period was therefore divided into a non-heating period (April, May, July, August, September, and October) and a heating period (November, December, and January) to explore the temporal variations in VOCs.

**Figure 3:**
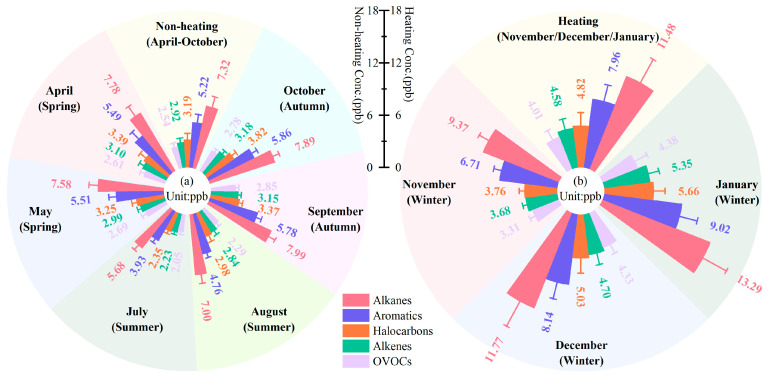
Characterization of temporal variations in VOC compositions in (**a**) non-heating and (**b**) heating periods during the observation period.

As shown in Figure 3, the concentration of TVOCs was 21.4 ± 3.7 ppb in the non-heating period, while in the heating period, it was about 1.6 times as high at 33.2 ± 8.5 ppb; alkanes, aromatics, and alkenes increased by 4.2 ppb, 2.7 ppb, and 1.7 ppb, respectively. Individual species such as n-butane, iso-butane, n-pentane, isopentane, n-octane, n-decane, n-undecane, n-dodecane, benzene, toluene, ethylbenzene, and naphthalene increased by more than 50%.

This is primarily attributable to relatively low winter temperatures, resulting in large-scale coal-fired heating by power stations and residents using biomass combustion for heating. In addition, during the periods of crop exports in autumn and import of winter supplies, traffic levels in Harbin peaked, and since the proportion of diesel vehicles was high, diesel vehicle exhausts contributed the most to the high levels of chain alkanes (>C_6_) and aromatics [54]. Furthermore, it takes longer for catalytic converters to reach their ignition temperature and work appropriately, meaning that VOCs are not burned completely in cold-start vehicles’ catalytic converters, resulting in much higher alkane and aromatic tracer emissions [22]. Nevertheless, 1,4-dichlorobenzene (−59.3%), vinyl acetate (−36.2%), and chlorotoluene (−7.2%) showed decreasing trends, probably because as solvent evaporation tracers, their evaporation decreases as the temperature drops.

Apart from prominent emission sources, temporal variations in VOC concentrations were associated with multiple factors, such as photochemical activity and meteorological conditions. VOCs were consumed in summer under conditions of high temperatures and strong UV radiation, whereas the high levels of VOCs in winter could often be attributed to sudden drops in temperature and lower boundary layers, which tend to form an inversion layer, which is unfavorable to the dilution and diffusion of VOCs.

**Figure 4:**
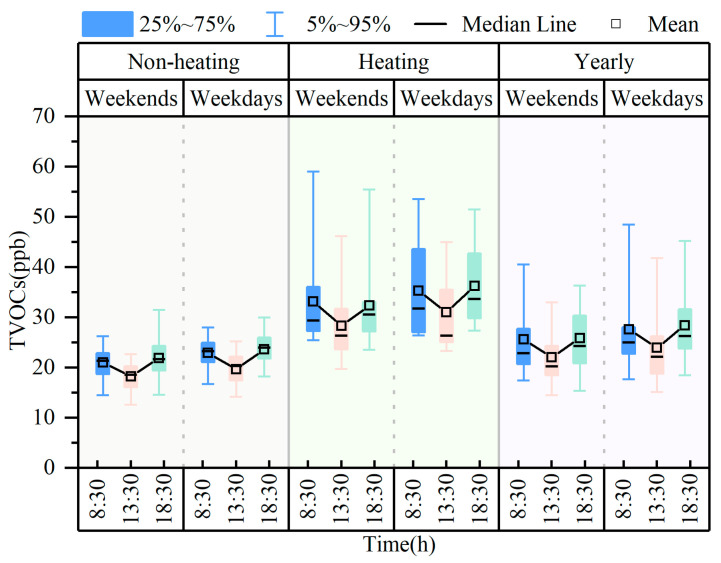
Weekend effects and daily variation in TVOCs.

Figure 4 shows the weekend effects and daily variation in TVOCs. The average TVOC concentrations for weekends and weekdays were 24.5 ± 7.9 ppb and 26.7 ± 8.3 ppb, respectively. Similar elevations in VOC concentrations on weekdays were found in studies in Shanghai, China [55], Sacramento [56], and Los Angeles [57], reflecting the lower anthropogenic activities on weekends leading to lower VOCs emissions.

The daily variation resulted in a “V” pattern of TVOCs within a day due to the combination of local anthropogenic emissions and meteorological conditions. High VOC values generally appeared in the morning, when the atmosphere was stable and the boundary layer was shallow, coupled with the strong influence of vehicle exhausts during the peak hours of morning (08:00–10:00 CST). In the afternoon, increased temperature, wind speed, and UV radiation, as well as an increased boundary layer, led to intense air convection, promoting photochemical consumption and diffusion of VOCs. With the arrival of late traffic peaks and unfavorable changes in meteorological conditions, VOC concentrations accumulated again and remained higher.

### 3.2. Source Apportionment

#### 3.2.1. Specific VOC Ratio

The ratio of VOC species with similar atmospheric lifetimes reflects the features of their sources, making it possible to use this as a basis for preliminarily distinguishing among emission sources.

**Figure 5:**
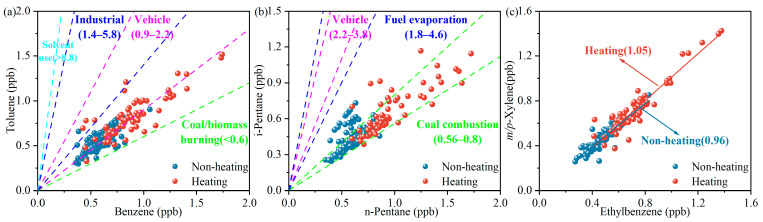
Specific ratios of (**a**) toluene to benzene, (**b**) i-pentane to n-pentane, and (**c**) *m*/*p*-xylene to ethylbenzene, used for source identification.

The toluene/benzene (T/B) ratio [58] is a widely used indicator for aromatics sources, ranging from <0.6, 0.9–2.2, 1.4–5.8, and >8.8 for combustion (coal and biomass), vehicle exhaust, industrial, and solvent usage sources, respectively. The average T/B ratio during the non-heating period was 0.9 ± 0.2 (*R_pearson_* = 0.75), with 40.8% of the values being within the range of 0.6–0.9 and 58.3% being within 0.9–1.4. The average T/B ratio during the heating period was 1.0 ± 0.2 (*R_pearson_* = 0.84), of which 93.9% were within the 0.6–1.4 range. These results implied that combustion emissions and vehicle exhaust sources might contribute significantly to VOC concentrations in the Harbin urban area (Figure 5a).

The ratio of i-pentane to n-pentane [59] is widely used as an indicator of combustion emissions, fuel evaporation, and vehicle exhaust sources, with this ratio ranging from 0.56 to 0.8, 1.8 to 4.6, and 2.2 to 3.8, respectively. In this study (Figure 5b), the average i/n-pentane ratio in the non-heating period was 0.8 ± 0.2 (*R_pearson_* = 0.51). A total of 77.8% were in the range of 0.56–0.8, indicating a significant influence of straw combustion in spring and autumn; moreover, 21.3% were in the range of 0.8–1.8, suggesting that fuel evaporation contributes to VOCs to some degree. During the heating period, the average i/n-pentane ratio was 0.7 ± 0.1 (*R_pearson_* = 0.83), with 91.7% being within the range of 0.56–0.8, demonstrating a significant contribution from coal-fired heating in winter.

As well as local emissions, regional transport is also a potential VOC source. M/p-xylene and ethylbenzene were found to be similar in terms of emission sources, yet the former exhibits 2.7–3.0 times more reactivity toward ·OH radicals than the latter. Lower ratios normally indicate greater air mass aging, i.e., that local VOCs are more influenced by external transport [55]; in this study, the X/E ratio was used to evaluate the impacts of regional transport. The linear correlation coefficient of the X/E ratio was 1.0 (*R_pearson_* = 0.91) in the non-heating period and 1.1 (*R_pearson_* = 0.94) in the heating period, as shown in Figure 5c, suggesting that more primary emissions came from anthropogenic sources during the heating period, while they may be influenced by the transport of smoke from straw burning from suburban and nearby cities during the non-heating period.

#### 3.2.2. PMF Method of Source Analysis

**Figure 6:**
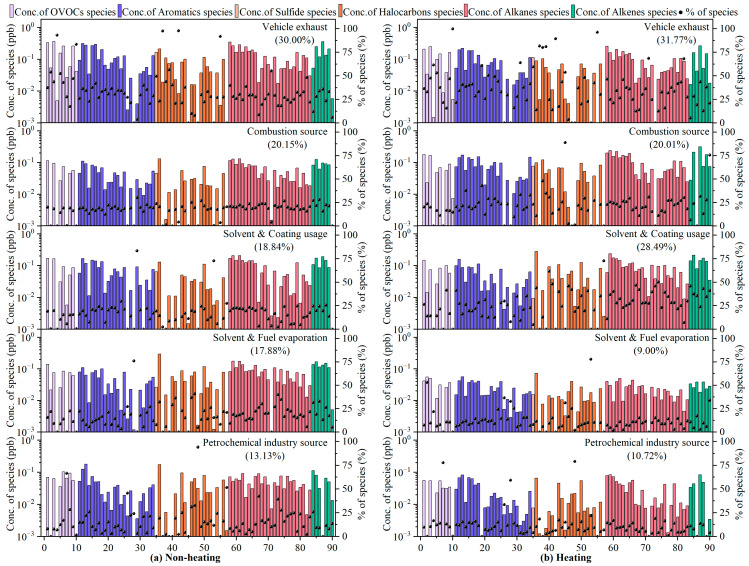
Source profiles and contributions of VOCs, identified using the PMF model. Bars represent the concentration of each species apportioned to the factor, and black dots represent the percentage of each species apportioned to the factor. The specific compounds corresponding to the values of the horizontal coordinates are detailed in Table S4.

In this study, 90 VOC species were put into the PMF model, which eventually resolved five potential emission sources: vehicle exhausts, combustion sources, solvent and coating usage, solvent and fuel evaporation, and petrochemical industry sources. The source profiles and contributions of VOCs that were identified using the PMF model are shown in Figure 6.

Factor 1 was enriched in iso-butane (46.5%), n-pentane (34.4%), cyclopentane (45.9%), 2-methylpentane (37.7%), 3-methylpentane (36.1%), 2,3-dimethylpentane (68.6%), n-octane (33.7%), nonane (41.9%), n-decane (43.4%), n-undecane (68.1%), 1-pentene (43.7%), MTBE (47.6%), benzene (34.3%), toluene (40.8%), m/p-xylene (41.1%), and 1,3,5-trimethylbenzene (49.4%). Gasoline vehicle exhaust is characterized by high proportions of low-carbon (C_2_–C_5_) alkanes and a certain percentage of aromatics (benzene, xylene, and trimethylbenzene) [54]. In addition, alkenes are strong indicators of incomplete combustion of gasoline [60]. MTBE is a well-known gasoline additive for fuel economy improvement [50]. Diesel vehicle exhaust is dominated by high-carbon alkanes (e.g., n-octane, n-decane, n-undecane), C_4_–C_5_ alkenes, and C_6_–C_9_ aromatics [54]. Taking this into consideration, Factor 1 was interpreted as vehicle exhaust emissions, which is consistent with the above T/B ratio results. On the other hand, by the end of 2021, the number of personal vehicles in Harbin exceeded 2.013 million [61]; this huge amount of vehicle holdings led directly to high vehicle emissions, resulting in a direct contribution of vehicle exhausts to VOCs of 31.8% and 30.0% in the non-heating and heating period, respectively.

Factor 2 was characterized by large loadings (20.2–88.8%) of toluene, naphthalene, methyl chloride, 1,1-dichloroethane, 1,1,2-trichloroethylene, carbon tetrachloride, iso-butane, n-butane, cyclohexane, n-heptane, cis-2-butene, trans-2-butene, isoprene, and 1-hexene. Studies have shown that open biomass burning could release significant amounts of chlorine-containing VOCs (e.g., methyl chloride, carbon tetrachloride) [62], aromatics (e.g., toluene, naphthalene) [63], and alkenes (e.g., isoprene emissions from smoldering rice straw fires) [64] into the atmosphere. Coal combustion is characterized by high concentrations of alkenes (e.g., 2-butene, 1-hexene) [65], lower carbon halocarbons (e.g., dichloroethane, trichloroethylene) [66], and alkanes below C_7_ (e.g., n-heptane, cyclohexane, hexane) [67]. In addition, n-butane and iso-butane may be derived from the fuel combustion of main engines that are powered by compressed natural gas (CNG) and liquefied petroleum gas (LPG) [68], with about 95% of the taxis in Harbin using CNG and LPG as fuel. To summarize, Factor 2 could be explained as having fossil fuel (coal, oil, natural gas) and biomass burning sources, which would also confirm the T/B and i-pentane/n-pentane ratios described above. In quantitative terms, non-heating-period combustion sources contributed 20.2% of VOCs, in large part due to open straw burning after the fall harvest and before the spring plowing, and 20.0% of VOCs during the heating period, which is attributed to coal-fired heating in winter.

Factor 3 was designated as solvent/coating usage sources because of the high abundance (19.6–83.3%) of acetone, ethyl acetate, tetrahydrofuran, toluene, ethylbenzene, xylene, styrene, 1,2,3-trimethylbenzene, 1,2,4-trimethylbenzene, perchloroethylene, trichloromethane, n-hexane, cyclohexane, methylcyclohexane, and n-octane. OVOCs (e.g., acetone, ethyl acetate), aromatics (e.g., toluene, ethylbenzene, xylene, trimethylbenzene, styrene), and C_6_–C_8_ alkanes are important indicators of industrial solvents, household solvents, and construction paint emissions [69,70]. Meanwhile, tetrahydrofuran [71] has been called a “universal solvent” and is widely used for surface coatings, anticorrosive coatings, printing inks, and so on. Dichloromethane and trichloromethane [72] are frequently employed as cleaning agents (e.g., for paint stripping) and for extraction and separation processes in industrial production. Perchloroethylene [52] is characterized as a dry-cleaning solvent. There was a significant difference in the contribution of solvents and paint usage sources to VOCs during the non-heating (18.8%) and heating (28.5%) periods. On the one hand, this could be attributed to the extensive use of adhesives and coatings for fixing and anticorrosive treatment of building insulation panels based on polystyrene resin in order to maintain heat in winter. On the other hand, the atmospheric lifetime of about ten days and lower reactivity of tracers such as acetone, toluene, xylene, n-hexane, and n-octane, together with extremely low temperatures and static weather conditions in winter, resulted in the accumulation of these source markers.

Key species of Factor 4 included chlorotoluene, 1,4-dichlorobenzene, methyl chloride, dichloromethane, trichloromethane, 1-butene, cis-2-butene, trans-2-butene, trans-1,3-dichloropropene, n-butane, iso-pentane, n-pentane, 3-methylpentane, 2,2,4-trimethylpentane, n-hexane, 2-methylhexane, 3-methylhexane, and n-heptane. Solvent evaporation can be identified by C_3_–C_5_ alkenes (e.g., 1-butene, trans-2-butene, and cis-2-butene) and some aromatics and halocarbons [73]. It has been thoroughly documented that iso-pentane and n-pentane are typically found in abundance in gasoline vapors due to their high vapor pressure [74], while C_5_–C_8_ alkanes such as 2,2,4-trimethylpentane are well-known gasoline additives for increasing the knock resistance of fuel [75], and iso-butane and n-butane are commonly associated with LPG/NG evaporation [76]. Hence, Factor 4 was identified as solvent and fuel volatilization sources, which contributed significantly more to VOCs in the non-heating period (17.9%) than in the heating period (9.0%), which is strongly related to the relatively high summer temperatures.

Factor 5 exhibited a high percentage of halocarbons (24.3–94.0%; e.g., trichloromethane, 1,1,1-trichloroethane, 1,1,2-trichloroethane, 1,1,2,2-tetrachloroethane), OVOCs (28.3–66.5%; e.g., 1,4-dioxane, methyl methacrylate), alkanes (20.5–51.6%; methylcyclopentane, n-heptane, 2,3,4-trimethylpentane, 2-methylheptane, 3-methylheptane, nonane, n-dodecane), aromatics (22.1–45.7%; e.g., toluene, chlorobenzene, benzyl chloride, styrene, 1,3-dichlorobenzene, 1,4-dichlorobenzene, diethylbenzene), and 1- butene (25.7%). Industrial emission sources are typically characterized by high levels of halocarbons—important raw materials for industrial production [77]. 1,4-dioxane, methyl methacrylate, n-heptane, 2-methylheptane, and 3-methylheptane are popular solvents in synthetic resins, rubber adhesives, paint thinners, wood furniture, and other types of industrial products [78,79]. Methylcyclopentane and 2,3,4-trimethylpentane are derived from petroleum products and released by evaporation from refineries [80]. Nonane and n-dodecane are key tracers for applications in asphalt industries [81]. Aromatics (benzene, toluene, and dichlorobenzene) are mostly used in industrial manufacturing and solvent production [82], while styrene is a signature pollutant in the coking industry [83]. Alkenes are by-products of steam cracking and catalytic cracking with crude oil and mainly associated with petroleum-related chemical production processes [84]. Consequently, we interpreted this factor as petrochemical industry emission sources, with the contribution to VOCs being 2.4% higher in the non-heating period than in the heating periods, perhaps due to the reduction in petrochemical production capacities caused by the epidemic closure in winter 2021.

### 3.3. Environmental Multi-Effect Assessment

#### 3.3.1. Contribution of Different VOCs’ Chemical Groups to Environmental Effects

**Figure 7:**
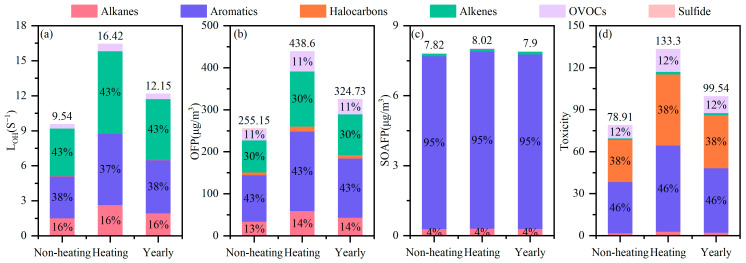
Contributions of different VOC chemical groups to environmental effects: (**a**) L_OH_, (**b**) OFP, (**c**) SOAFP, and (**d**) toxicity.

The atmospheric VOCs, due to their photochemical reactivity, could not only react with ·OH and NO_x_ radicals to generate O_3_ but were also closely associated with the formation of new particles, which triggered the discussion on SOAFP. This is why we must assess the different environmental effects of VOCs from a multidimensional perspective. Figure 7 illustrates the contributions of different VOC chemical groups to their environmental effects.

The average VOC loss rate due to reaction with ·OH was 12.2 ± 4.6 s^−1^ during the observation period and about 1.7 times higher in the heating period (16.4 ± 4.5 s^−1^) than in the non-heating period (9.5 ± 1.8 s^−1^). Alkenes and aromatics had the highest L_OH_, contributing around 42.7% and 37.6%, respectively. Under the combined conditions of significant photochemical reactivity and high concentration, cis-2-butene, trans-2-butene, 1-pentene, isoprene, cis-2-pentene, trans-2-pentene, xylene, styrene, trimethylbenzene, and naphthalene made the greatest contributions to the total L_OH_, amounting to 69.5%, which is generally in agreement with studies conducted in Xi’an [58] and Lhasa [31] cities.

The OFP was calculated to be 255.2 ± 47.1 µg/m^3^ during the non-heating period, compared with an increase of 71.9% during the heating period (438.6 ± 120.0 µg/m^3^), implying that increased anthropogenic emissions during winter lead to greater potential environmental effects. This represents another method for quantitatively assessing the contribution of VOCs to O_3_ generation, yielding similar results for the contributions of different VOC chemical groups to L_OH_. While the concentrations of aromatics and alkenes were lower than that of alkanes (alkanes accounted for 34.4% of TVOCs but contributed 13.5% to OFP), their contributions to OFP were the most significant, at 43.0% and 29.9%, respectively, suggesting that they held key roles in perturbing the photochemistry of O_3_. Specifically, methyl methacrylate had the highest OFP among the OVOCs of 20.2 ± 10.6 µg/m^3^, while toluene, ethylbenzene, xylene, m/p-ethyltoluene, trimethyltoluene, naphthalene, 1-butene, cis-2-butene, trans-2-butene, 1-pentene, isoprene, cis-2-pentene, and trans-2-pentene together contributed 65.4%, which was similar to findings obtained in Shanghai [55].

As the major secondary component of PM_2.5_, SOA is an important culprit in haze formation. The SOAFP was 7.9 ± 1.0 µg/m^3^ during our measurement period. This level was considerably higher than those found in Beijing [59], Shanghai [55], and Nanjing [85], China, which may be related to the number of VOCs, since we monitored about twice as many VOCs in this study as the above mentioned studies and other sources. SOAFP accounted for 23.3% of the PM_2.5_ concentration (SOAFP value/PM_2.5_ value), which was relatively consistent with the actual monitoring results in Harbin during 2018–2021 obtained by Cheng et al. [86]. Aromatics were identified as the most dominant contributor to SOAFP with 94.7%, while benzene, toluene, n-propylbenzene, iso-propylbenzene, xylene, and styrene were the most important VOC species for SOA formation, which was in agreement with urban studies conducted in Jinan [87], Shanghai [55], and Nanjing [85]. Consequently, Harbin should prioritize controlling aromatics to alleviate haze pressure.

Along with their indirect environmental harm, some VOC species have direct toxic effects. Our findings revealed that the average toxic effect of VOCs was 99.5 ± 37.5. Compared with the non-heating period (78.9 ± 15.8), this increased in the heating period (133.3 ± 38.2) by 68.9%. This may be related to anthropogenic emissions, such as coal-fired heating and insufficient combustion of vehicle exhaust in winter, leading to substantial rises in the concentrations of aromatics (by 52.5%), halocarbons (by 51.1%), and OVOCs (by 57.9%). Aromatics (represented by benzene, toluene, ethylbenzene, xylene, naphthalene, etc.), halocarbons (represented by vinyl chloride, methylene chloride, trichloromethane, 1,2-dichloropropane, tetrachloroethylene, etc.), and OVOCs (represented by MTBE and 4-methyl-2-pentanone) contributed more than 95.0%, which was comparable to the results from Ye et al. [31] and Niu et al.’s [21] investigations.

#### 3.3.2. Contributions of Different VOC Sources to Environmental Effects

Identifying source-specific contributions is clearly significant for targeted mitigation of negative environmental effects. Taking our PMF source apportionment results into account, the detailed contributions of different VOC sources to various environmental effects were calculated.

**Figure 8:**
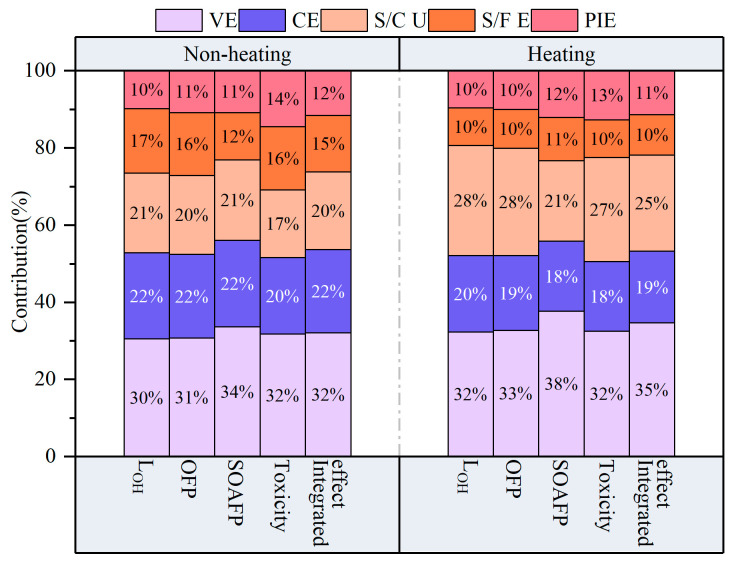
Contributions of different VOC sources to environmental effects (L_OH_, OFP, SOAFP toxicity, and integrated effects). VE: vehicle exhaust; CE: combustion source; S/C U: solvent and coating usage; S/F E: solvent and fuel evaporation; PIE: petrochemical industry source.

As shown in Figure 8, vehicle exhausts contributed the most to negative environmental effects (L_OH_, OFP, SOAFP, and toxicity), accounting for 32.0% and 34.6% of integrated effects in the non-heating and heating period, respectively, followed by combustion sources, which contributed 20.2% on average. Compared with the heating period (10.5%), solvent and fuel evaporation contributed 14.7% to integrated environmental effects during the non-heating period, whereas solvent and coating usage contributed 4.8% less compared with the heating period. This was in good accordance with the previous source analysis results, suggesting that increased anthropogenic emissions produce greater environmental impacts. As a result, in order to mitigate the occurrence of haze and other pollution phenomena in Harbin, controlling vehicle exhausts, combustion sources, and solvent and paint usage should be prioritized.

### 3.4. Human Health Risk Assessment

#### 3.4.1. Non-Carcinogenic Risk Exposure Assessment of Hazardous VOCs

Non-carcinogenic exposure risk explains the dose–response relationship for non-carcinogenic health effects and is used to estimate the probability of triggering non-carcinogenic adverse health effects during a given exposure period [34].

**Figure 9:**
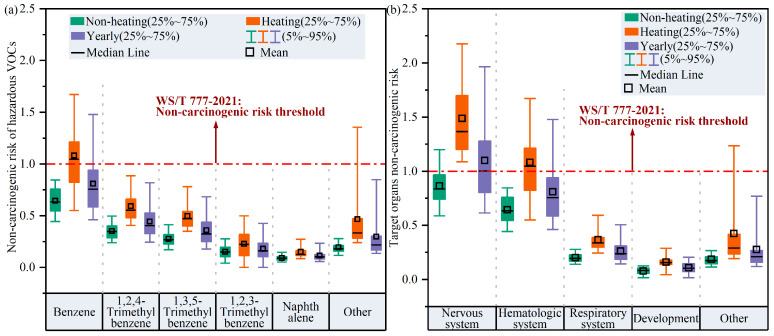
(**a**) Non-carcinogenic risk exposure assessment of hazardous VOCs and (**b**) non-carcinogenic risk assessment for target organs due to hazardous VOCs. In panel (**a**), “other” includes isopropanol, MTBE, vinyl acetate, 1,4-dioxane, toluene, chlorobenzene, ethylbenzene, o/m/p-xylene, styrene, 1,4-dichlorobenzene, carbon disulfide, tetrafluoroethylene dichloride, monobromomethane, ethylene chloride, monofluorotrichloromethane, 1,1-dichloroethylene, methylene chloride, trichloromethane, 1,1,1-trichloroethane, carbon tetrachloride, 1,1,2-tetrachloroethylene, tetrachloroethylene, 1,2-dibromoethylene, tetrachloroethylene, and n-hexane. In panel (**b**), “other” includes kidney, eyes, alimentary system, cardiovascular system, cardiovascular system, endocrine system, bones, and teeth.

Figure 9 shows the non-carcinogenic risk exposure assessment for hazardous VOC species and their corresponding target organs in Harbin’s atmosphere. Among the 32 hazardous VOCs, benzene (HQ = 1.1 ± 0.3; 5~95%: 0.6–1.7) presented the highest non-carcinogenic risk during the heating period, and 54.7% of these HQ values exceeded the threshold of 1 that is recommended by the National Health Commission of China (WS/T 777-2021) [37], demonstrating that there was a certain non-carcinogenic risk in Harbin due to benzene in the atmosphere during the heating period. In contrast, the HQ for benzene in the non-heating period was 0.4–0.8 (5~95%), with an average of 0.6 ± 0.1, indicating a comparatively minimal non-cancer risk from benzene during the non-heating period. In addition, the HQ values for trimethylbenzene and naphthalene were lower than the safety threshold of 1 during both the heating and non-heating period; therefore, we may infer that the non-carcinogenic threats to public health from these hazardous VOCs were essentially negligible.

Based on the fact that individual VOCs could potentially harm different organs, we further evaluated the non-carcinogenic risks from hazardous VOCs to different target organs. Compared with the non-heating period, the nervous system (HI = 1.5 ± 0.4; 5~95%: 1.1–2.2) and hematologic system (HI = 1.1 ± 0.3; 5~95%: 0.6–1.7) were exposed to significantly greater non-carcinogenic risks during the heating period, possibly urging more attention to mitigating non-carcinogenic risks faced by the nervous and hematological systems during heating periods.

#### 3.4.2. Carcinogenic Risk Exposure Assessment of Hazardous VOCs

Carcinogenic risk assessment is used to quantitatively characterize the relationship between exposure (dose) to hazardous VOCs and cancer incidence (response), making carcinogenic risk a significant concern [34].

**Figure 10:**
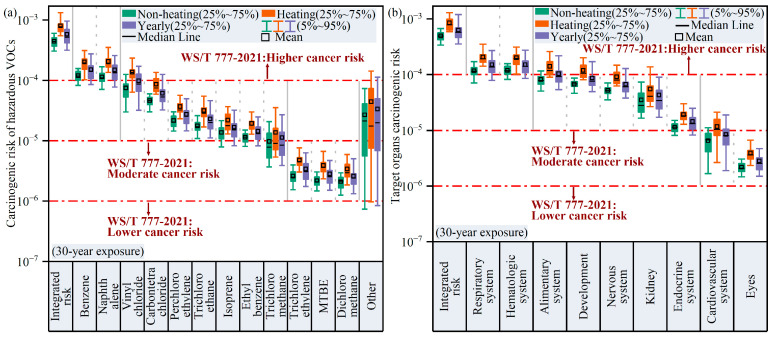
(**a**) Carcinogenic risk exposure assessment of hazardous VOCs for 30-year exposure scenario and (**b**) carcinogenic risk assessment for target organs due to hazardous VOCs for 30-year exposure scenario. In panel (**a**), “other” includes 1,4-dioxane, chlorotoluene, 1,4-dichlorobenzene, 1,1-dichloroethane, 1,2-dibromoethane, and 1,1,2,2-tetrachloroethane.

We assessed the carcinogenic risk of 18 carcinogens in Harbin using three exposure scenarios (9-year, 30-year, and 70-year exposure durations) (Figure 10 and Figure S4), which corresponded to the estimation of average, high-end, and lifetime residency exposures, respectively. The calculated average integrated cancer risk (ICR) from inhalation of hazardous VOCs for the 70-year exposure scenario was 6.8 × 10^−4^ (95% CI: 6.4 × 10^−4^–7.2 × 10^−4^), which was significantly higher than those estimated for the 30-year (ICR: 5.8 × 10^−4^; 95% CI: 5.4 × 10^−4^–6.1 × 10^−4^) and 9-year (ICR: 4.1 × 10^−4^; 95% CI: 3.9 × 10^−4^–4.4 × 10^−4^) exposure scenarios. The ICRs of carcinogenic VOCs under these three scenarios greatly exceeded the higher cancer risk level set by WS/T 777-2021 (1.0 × 10^−4^) [37], implying a significant health concern due to carcinogenic VOCs in the area.

Since the 30-year exposure duration is recommended in the OEHHA guidance as a baseline for high-end cancer risk assessments, all cancer-associated risks presented hereafter are estimates from the 30-year exposure scenario [34]. The carcinogenic risks of benzene and naphthalene were found to be 1.51 × 10^−4^ (5~95%: 8.6 × 10^−5^–2.8 × 10^−4^) and 1.48 × 10^−4^ (5~95%: 7.8 × 10^−5^–2.6 × 10^−4^), indicating that high concentrations of benzene and naphthalene were the most hazardous to human health of the aromatic VOCs species, which agrees with the conclusions in the study by Gao et al. [23], which was also conducted in Harbin. We particularly emphasize that benzene and naphthalene may be significant risk factors, mostly due to their high toxicity (benzene and naphthalene have CPFs of 0.1 and 0.12, respectively), as well as their widespread sources and significant emissions. Consequently, we reiterate that carcinogenic exposure due to benzene and naphthalene should be given urgent attention. Additionally, vinyl chloride, carbon tetrachloride, tetrachloroethylene, trichloroethane, isoprene, ethylbenzene, and trichloromethane exhibited carcinogenicity risks between the moderate (1.0 × 10^−5^) and higher (1.0 × 10^−4^) cancer risks recommended in WS/T 777-2021 [37], emphasizing that sufficient priority should be given to these.

The carcinogenic risk assessment for target organs indicated that the hematologic system (CR = 1.5 × 10^−4^; 5~95%: 8.6 × 10^−5^–2.8 × 10^−4^), respiratory system (CR = 1.5 × 10^−4^; 5~95%: 7.9 × 10^−5^–2.7 × 10^−4^), and alimentary system (CR = 1.0 × 10^−4^; 5~95%: 5.4 × 10^−5^–2.2 × 10^−4^) were all exposed to higher carcinogenic risks, necessitating greater attention to cardiovascular diseases, airway diseases, intestinal diseases, etc., which are associated with exposure to hazardous VOCs.

Nevertheless, this conclusion is subject to some limitations owing to constraints on activity hours in outdoor environments, considering that residents spent 90% of their time in indoor environments, especially in winter when temperatures were extremely low, resulting in a lower air exchange rate; hence, the health risk from VOCs in the outdoor atmosphere may be overestimated.

#### 3.4.3. Contributions of Different VOC Sources to Health Risks

**Figure 11:**
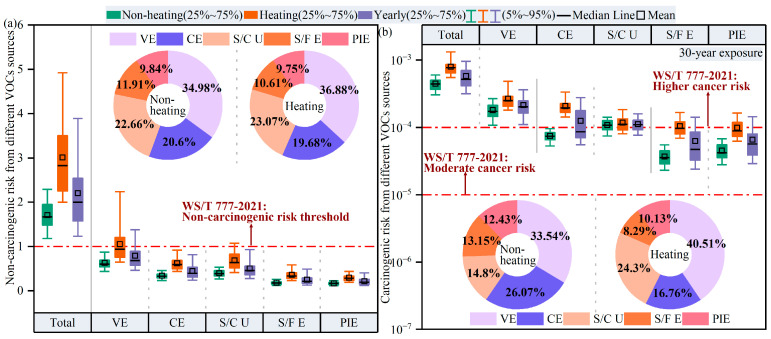
Contributions of different VOC sources to (**a**) non-carcinogenic and (**b**) carcinogenic risks.

Apart from individual VOC species, as shown in Figure 11, the contribution of five key VOC sources to health risks in Harbin was evaluated by conducting cumulative risk calculations of hazardous VOCs from each source.

According to our results, five VOC sources contributed to non-carcinogenic and carcinogenic risks with similar regularity. During the heating period, vehicle exhaust and solvent and coating usage contributed 36.9% and 23.1% to the non-carcinogenic risk and reached 40.5% and 24.3% contributions to the carcinogenic risk. During the non-heating period, combustion sources made a contribution of 20.6% to the non-carcinogenic risk, while their contribution to the carcinogenic risk reached 26.1%. Consequently, vehicle exhaust, combustion sources, and solvent and coating usage posed the greatest threats to human health. In combination with our findings in Section 3.2.2, it can be seen that aromatics, represented by benzene and naphthalene, were common tracers for these three sources, which again supports our findings of higher health risks from benzene, naphthalene, and other VOC species, presented in Section 3.4.1 and Section 3.4.2. Therefore, to minimize adverse health outcomes associated with exposure to hazardous VOCs, it is imperative to control emissions from anthropogenic sectors across Harbin.

## 4. Conclusions

Since the estimated cancer risks due to inhalation of hazardous VOCs such as benzene and naphthalene consistently exceeded acceptable risk levels (Figure 10a), we calculated the PRGs and associated reduction rates for these priority VOCs in the atmosphere of Harbin. As demonstrated in Table 1, during non-heating periods, benzene and naphthalene concentrations must be reduced by 16.5% and 12.1% to achieve the cancer risk levels recommended by WS/T 777-2021, whereas during heating periods, the rate of reduction must be above 50.0%. In addition, the concentration of Vinyl chloride would need to decrease by 20.9% during heating periods to meet the suggested standard of 1.0 × 10^−4^. Smaller reductions were achieved for benzene, naphthalene, and Vinyl chloride due to their higher cancer risks, yet greater efforts remain necessary to reduce the ambient levels of these compounds to reach moderate or low cancer risks (1.0 × 10^−5^ or 1.0 × 10^−6^), as this would demand emission reductions exceeding 90.0%. The PRGs of individual VOC sources in Harbin were also estimated, indicating that 78.5% and 50.7% reductions in vehicle exhausts and solvent and coating usage are required during non-heating periods, while 82.6%, 40.7%, and 12.9% reductions in vehicle exhausts, combustion sources, and solvent and fuel evaporative emissions, respectively, should be implemented during heating periods to prevent cancer-associated risks. Considering the abundance of benzene and naphthalene in these four source categories, the significant reductions in concentrations that are required for these compounds is not surprising. These calculated values provide preliminary goals for policy makers to prioritize VOC control strategies in Harbin. Accordingly, significant joint environmental and health benefits can be expected from cutting key anthropogenic emissions in Harbin.

This study investigated the characterization of VOCs, their different sources, and their associated environmental and health risks during non-heating and heating periods. It should be noted that there are several uncertainties associated with our risk assessment and PMF modeling, which can be minimized in future studies by reducing the measurement uncertainties of exposure variables, thus improving the representation of VOC data, and performing diffusion-normalized PMF modeling. Despite these limitations, our employment of a health risk-oriented source apportionment approach still has significant implications for formulating tailored air pollution mitigation policies to achieve both environmental and health-related benefits. This study represents a significant step forward towards quantifying emission reduction goals for important VOC species and sources, which could support local air quality control and health awareness.

## Figures and Tables

**Table 1:** Estimated reduction rates for hazardous VOCs and their sources in Harbin based on a 30-year exposure scenario ^a^.

		Non-Heating			Heating		
		1.0 × 10^−6^	1.0 × 10^−5^	1.0 × 10^−4^	1.0 × 10^−6^	1.0 × 10^−5^	1.0 × 10^−4^
VOC species	MTBE	54.36%			74.45%		
	1,4-Dioxane	77.34%			87.63%		
	Benzene	99.16%	91.65%	16.45%	99.50%	95.04%	50.35%
	Ethylbenzene	91.25%	12.45%		94.82%	48.23%	
	Chlorotoluene	89.28%			90.18%	1.76%	
	1,4-Dichlorobenzene	74.00%			43.12%		
	Naphthalene	99.12%	91.21%	12.13%	99.51%	95.12%	51.17%
	Vinyl chloride	98.40%	83.97%		99.21%	92.09%	20.88%
	Dichloromethane	52.50%			70.78%		
	Trichloromethane	89.77%			92.74%	27.43%	
	Carbon tatrachloride	97.84%	78.38%		98.84%	88.40%	
	1,1,2-Trichloroethylene	61.55%			79.05%		
	1,1,2-Trichloroethane	94.33%	43.29%		96.84%	68.43%	
	1,2-Dibromoethane	14.45%			93.22%	32.17%	
	Perchloroethylene	95.37%	53.75%		97.28%	72.81%	
	1,1,2,2-Tetrachloroethane	73.24%			87.67%		
	Isoprene	92.74%	27.43%		95.44%	54.42%	
VOC sources	Vehicle exhaust	99.78%	97.85%	78.46%	99.83%	98.26%	82.61%
	Combustion source	98.41%	84.13%		99.41%	94.07%	40.73%
	Solvent and coating usage	99.51%	95.07%	50.74%	98.81%	88.12%	
	Solvent and fuel evaporation	96.07%	60.67%		99.13%	91.29%	12.88%
	Petrochemical industry source	97.58%	75.84%		99.23%	92.30%	

^a^ Hazardous VOCs and key source reduction rates were calculated based on WS/T 777-2021 recommendations for lower, moderate, and higher cancer risk levels using the methodology described in Section 2.4.3. The blank cell indicates that the associated cancer risk level has not been exceeded; thus, no reduction rate is required to be calculated.

## Data Availability

Data will be made available upon request.

## Supplementary Materials

The following supporting information can be downloaded at https://www.mdpi.com/article/10.3390/toxics14010046/s1, Figure S1: The locations of the sampling site (in red star) in Harbin. Figure S2. Time series of mete-orological parameters and levels for air pollutants during the sampling period. Figure S3. Correla-tion Analysis Between Meteorological Parameters and Air Pollutant Concentrations. Figure S4. (a) Carcinogenic risk exposure assessment of hazardous VOCs for 9—year exposure scenarios, and (b) carcinogenic risk exposure assessment of hazardous VOCs for 70—year exposure scenarios. Table S1: Detailed information about sampling time. Table S2. The target VOCs basic information with MDL, RSD and R2 [88,89]. Table S3. Calculation parameters related to the environment intergrated effects assessment of VOCs [21,26,27,28,29,30,90]. Table S4. VOCs species included in PMF source appor-tionment modeling. Table S5. Summary of the PMF error estimation diagnostics using VOCs dataset in Harbin. Table S6. Mapping of BS factors to the base factors at a 5-factor solution for Non-heating dataset. Table S7. Mappinga of BS factors to the base factors at a 5-factor solution for Heating da-taset. Table S8. The REL and CPF values of the selected hazardous VOCs and target organs [34]. Table S9. Recommended parameter values for cancer risk calculation for different age groups [34].
