# Supplementary material for: Environmental and Health Risk Assessments of Volatile Organic Compounds (VOCs) Based on Source Apportionment—A Case Study in Harbin, a Megacity in Northeastern China
Source: Toxics. 2025 Dec 31;14(1):46. doi: 10.3390/toxics14010046 (PMC12845896; doi:10.3390/toxics14010046)
Supplement: Supplementary file 1 [file toxics-14-00046-s001.zip › Toxics-support information.pdf]

Article

Environmental and Health Risk Assessments of Volatile Organic Compounds (VOCs) Based on Source Apportionment—A Case Study in Harbin, a Megacity in Northeastern China

Jinpan Jiang [1,2], Bo Li [3], Binyuan Wang [1,2], Lu Lu [1,2], Fan Meng [1,2], Chongguo Tian [4], Hong Qi [1,2,*] and Ai-Ling Lian [5,*]

1 School of Environment, Harbin Institute of Technology, Harbin 150090, China
2 State Key Laboratory of Urban-Rural Water Resource and Environment, Harbin Institute of Technology, Harbin 150090, China
3 School of Environment, Tsinghua University, Beijing 100084, China
4 Key Laboratory of Coastal Environmental Processes and Ecological Remediation, Yantai Institute of Coastal Zone Research, Chinese Academy of Sciences, Yantai 264003, China
5 Department of Operating Room, First Affiliated Hospital of Harbin Medical University, Harbin 150001, China
* Correspondence: hongqi@hit.edu.cn (H.Q.); lianailing@hrbmu.edu.cn (A.-L.L.)

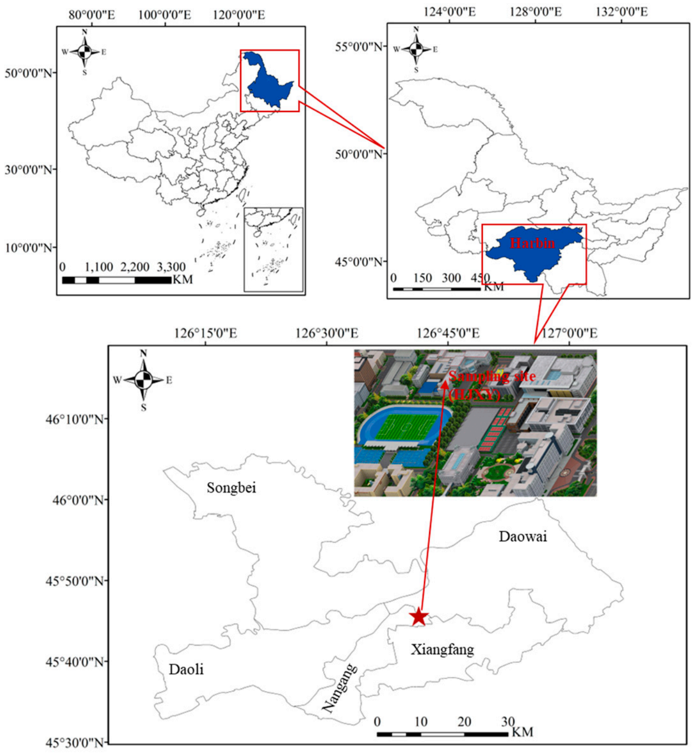

Figure S1. The locations of the sampling site (in red star) in Harbin.

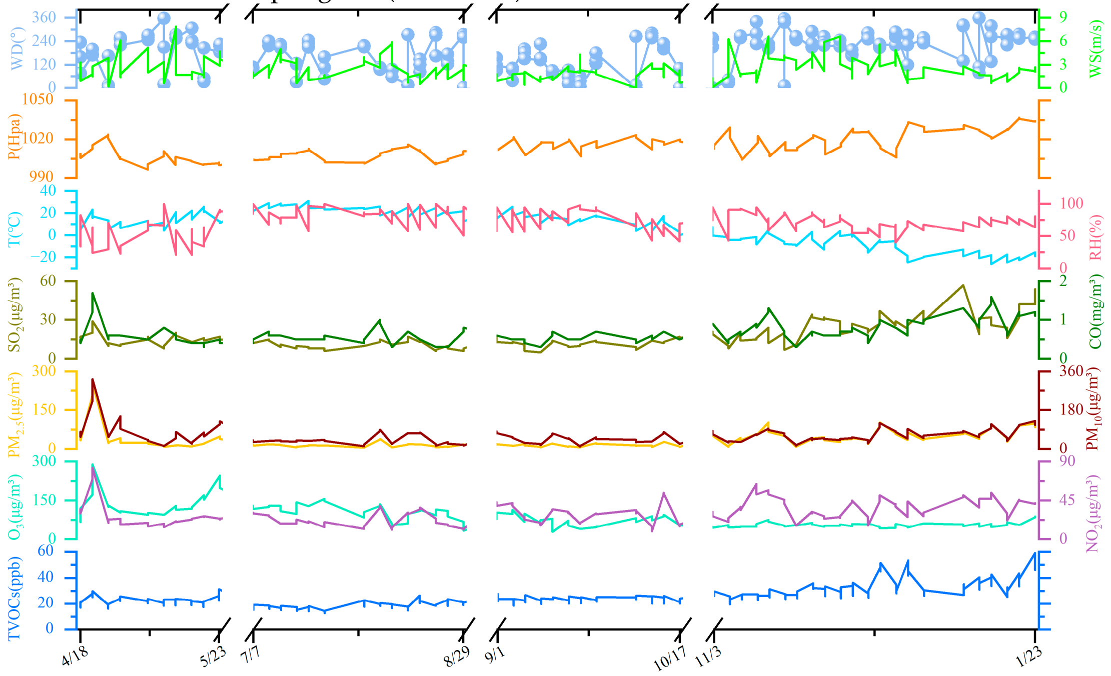

Figure S2. Time series of meteorological parameters and levels for air pollutants during the sampling period.

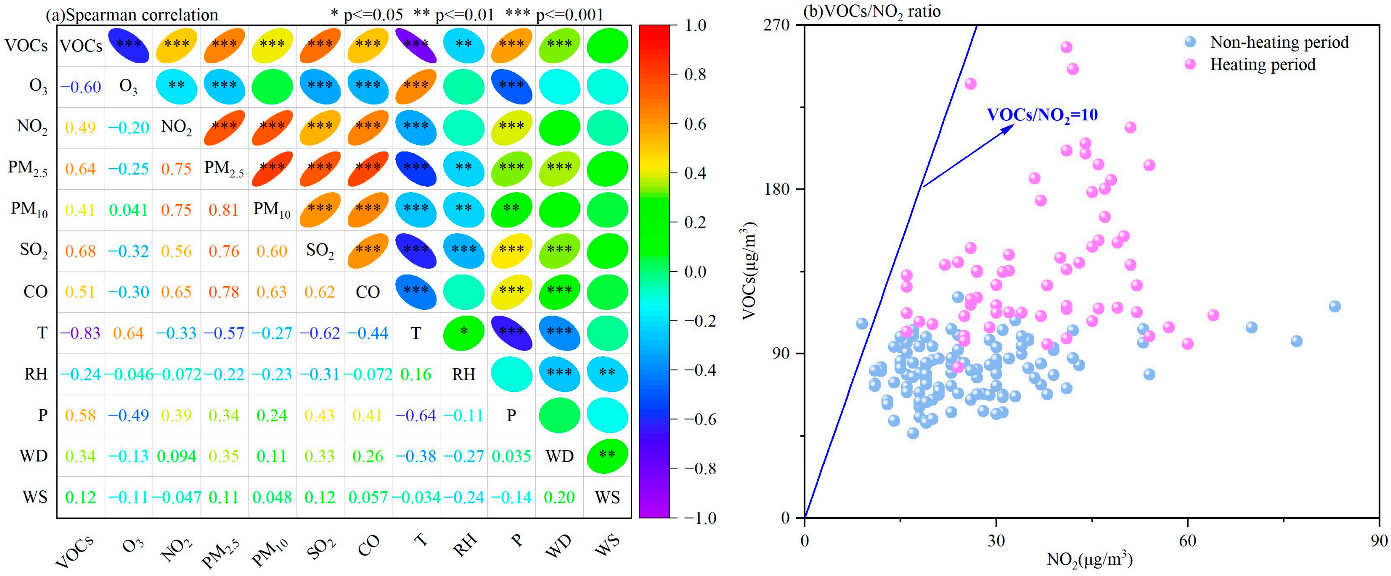

Figure S3. Correlation Analysis Between Meteorological Parameters and Air Pollutant Concentrations.

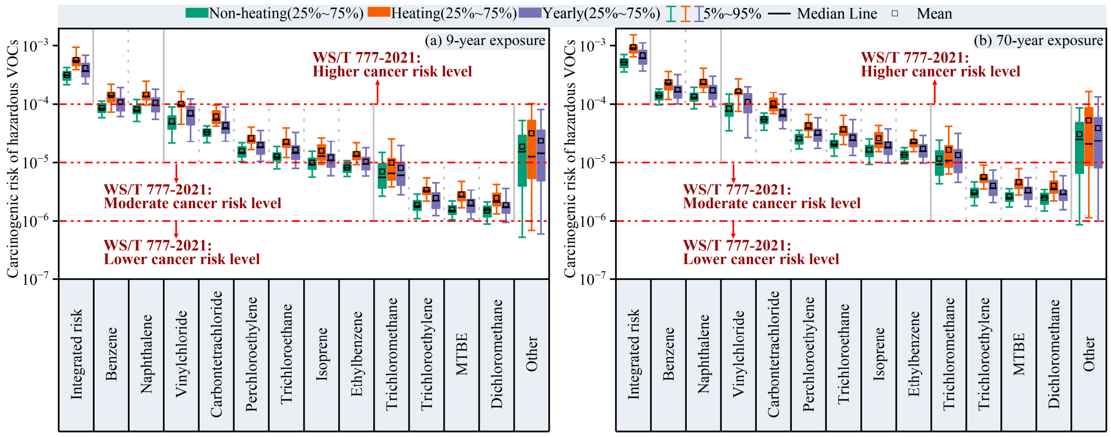

Figure S4. (a) Carcinogenic risk exposure assessment of hazardous VOCs for 9 - year exposure scenarios, and (b) carcinogenic risk exposure assessment of hazardous VOCs for 70 - year exposure scenarios.

Table S1. Detailed information about sampling time.

H or Non-h[a]	Seasons	Months	Date	Time[b]	Numbers[c]
Non-h	Spring	4	18/21/25/28	8:30/13:30/18:30	12
		5	5/9/12/16/19/23	8:30/13:30/18:30	18
	Summer	7	7/11/14/18/21/25	8:30/13:30/18:30	18
		8	4/8/11/15/18/22/25/29	8:30/13:30/18:30	24
	Autumn	9	1/5/8/12/15/19/22/26	8:30/13:30/18:30	24
		10	6/10/13/17	8:30/13:30/18:30	12
H	Winter	11	3/7/10/14/17/21/24/28	8:30/13:30/18:30	24
		12	1/5/8/12/15/19/22/26	8:30/13:30/18:30	24
		1	5/9/12/16/19/23	8:30/13:30/18:30	18

[a] **H or Non-h:** H for heating period and Non-h for non-heating period.

[b] **Time:** China Standard Time (CST).

[c] **Numbers:** Number of samples.

Table S2. The target VOCs basic information with MDL, RSD and R^2.

No.	Species	CAS	Molar.[b]	P or T[c]	MDL	RSD	R^2
**Alkanes**							
1	iso-Butane	75-28-5	58.12	P	0.038	3%	0.995
2	n-Butane	106-97-8	58.12	P	0.038	5%	0.997
3	iso-Pentane	78-78-4	72.15	P	0.025	6%	0.999
4	n-Pentane	109-66-0	72.15	P	0.028	4%	0.994
5	2,2-Dimethylbutane	75-83-2	86.18	P	0.007	7%	0.993
6	Cyclopentane	287-92-3	70.13	P	0.030	4%	0.991
7	**2,3-Dimethylbutane[a]**	79-29-8	86.18	P	0.006	6%	0.990
8	**2-Methylpentane[a]**	107-83-5	86.18	P	0.006	7%	0.990
9	3-Methylpentane	96-14-0	86.18	P	0.006	5%	0.993
10	n-Hexane	110-54-3	86.18	T/P	0.006	5%	0.995
11	Methylcyclopentane	96-37-7	84.16	P	0.005	5%	0.991
12	2,4-Dimethylpentane	108-08-7	100.20	P	0.004	5%	0.995
13	Cyclohexane	110-82-7	84.16	T/P	0.006	5%	0.993
14	2-Methylhexane	591-76-4	100.20	P	0.005	5%	0.997
15	2,3-Dimethylpentane	565-59-3	100.20	P	0.008	4%	0.997
16	3-Methylhexane	589-34-4	100.20	P	0.003	6%	0.997
17	2,2,4-Trimethylpentane	540-84-1	114.23	P	0.003	5%	0.996
18	n-Heptane	142-82-5	100.20	T/P	0.005	6%	0.995
19	Methylcyclohexane	108-87-2	98.19	P	0.007	6%	0.995
20	2,3,4-Trimethylpentane	565-75-3	114.23	P	0.006	6%	0.995
21	2-Methylheptane	592-27-8	114.23	P	0.008	6%	0.994
22	3-Methylheptane	589-81-1	114.23	P	0.007	6%	0.996
23	n-Octane	111-65-9	114.23	P	0.004	6%	0.997
24	Nonane	111-84-2	128.26	P	0.004	6%	0.998
25	n-Decane	124-18-5	142.28	P	0.006	4%	0.998
26	n-Undecane	1120-21-4	156.31	P	0.005	4%	0.995
27	n-Dodecane	112-40-3	170.33	P	0.005	4%	0.995

**Alkenes**							
28	1-Butene	106-98-9	56.11	P	0.026	5%	0.993
29	trans-2-Butene	624-64-6	56.11	P	0.029	6%	0.992
30	cis-2-Butene	590-18-1	56.11	P	0.027	3%	0.992
31	1-Pentene	109-67-1	70.13	P	0.012	7%	0.994
32	Isoprene	78-79-5	68.12	P	0.009	6%	0.996
33	**trans-2-Pentene**[a]	646-04-8	70.13	P	0.006	5%	0.993
34	**cis-2-Pentene**[a]	627-20-3	70.13	P	0.006	5%	0.993
35	1-Hexene	592-41-6	84.16	P	0.008	5%	0.993
**Halocarbons**							
36	Methyl chloride	74-87-3	50.49	T	0.004	5%	0.994
37	Dichlorotetrafluoroethane	76-14-2	170.92	T	0.004	5%	0.997
38	Vinyl chloride	75-01-4	62.50	T	0.008	5%	0.995
39	Bromomethane	74-83-9	94.94	T	0.008	4%	0.996
40	Chloroethane	75-00-3	64.51	T	0.006	4%	0.997
41	Trichlorofluoromethane	75-69-4	137.37	T	0.007	6%	0.997
42	1,1-Dichloroethene	75-35-4	96.94	T	0.005	6%	0.997
43	Methylene chloride	75-09-2	84.93	T	0.005	6%	0.997
44	trans-1,2-Dichloroethylene	156-60-5	96.94	T	0.004	6%	0.998
45	1,1-Dichloroethane	75-34-3	98.96	T	0.005	6%	0.997
46	cis-1,2-Dichloroethylene	156-59-2	96.94	T	0.005	6%	0.997
47	Trichloromethane	67-66-3	119.38	T	0.006	6%	0.995
48	1,1,1-Trichloroethane	71-55-6	133.40	T	0.006	6%	0.991
49	Tetrachloromethane	56-23-5	153.82	T	0.005	4%	0.992
50	1,2-Dichloropropane	78-87-5	112.99	T	0.005	4%	0.993
51	Bromodichloromethane	75-27-4	163.83	T	0.006	5%	0.995
52	Trichloroethylene	79-01-6	131.39	T	0.002	5%	0.996
53	cis-1,3-Dichloropropene	10061-01-5	110.97	T	0.004	5%	0.997
54	trans-1,3-Dichloropropene	10061-02-6	110.97	T	0.004	5%	0.997
55	1,1,2-Trichloroethane	79-00-5	133.40	T	0.007	5%	0.991
56	Dibromochloromethane	124-48-1	208.28	T	0.005	5%	0.990
57	1,2-Dibromoethane	106-93-4	187.86	T	0.005	5%	0.992
58	Tetrachloroethene	127-18-4	165.83	T	0.006	6%	0.992
59	Tribromomethane	75-25-2	252.73	T	0.005	6%	0.993
60	1,1,2,2-Tetrachloroethane	79-34-5	167.85	T	0.006	6%	0.995
61	Hexachloro-1,3-butadiene	87-68-3	260.76	T	0.005	6%	0.995
**Aromatics**							
62	Tetrahydrofuran	109-99-9	72.11	T	0.007	3%	0.995
63	Benzene	71-43-2	78.11	T/P	0.004	6%	1.000
64	Toluene	108-88-3	92.14	T/P	0.004	7%	0.998
65	Chlorobenzene	108-90-7	112.56	T	0.005	2%	0.996
66	Ethylbenzene	100-41-4	106.17	T/P	0.007	5%	0.997
67	**m-Xylene**[a]	108-38-3	106.17	T/P	0.007	4%	0.996

68	**p-Xylene**[a]	106-42-3	106.17	T/P	0.007	6%	0.996
69	Styrene	100-42-5	104.15	T/P	0.006	6%	0.995
70	o-Xylene	95-47-6	106.17	T/P	0.006	6%	0.995
71	Isopropylbenzene	98-82-8	120.19	P	0.008	6%	0.995
72	n-Propylbenzene	103-65-1	120.19	P	0.006	6%	0.995
73	m-Ethyltoluene	620-14-4	120.19	P	0.009	6%	0.997
74	4-Ethyltoluene	622-96-8	120.19	T/P	0.008	6%	0.994
75	1,3,5-Trimethylbenzene	108-67-8	120.19	T/P	0.008	4%	0.997
76	o-Ethyltoluene	611-14-3	120.19	P	0.005	5%	0.997
77	1,2,4-Trimethylbenzene	95-63-6	120.19	T/P	0.008	4%	0.992
78	Benzyl chloride	100-44-7	126.58	T	0.010	4%	0.996
79	1,3-Dichlorobenzene	541-73-1	147.00	T	0.007	4%	0.994
80	1,4-Dichlorobenzene	106-46-7	147.00	T	0.007	4%	0.995
81	1,2,3-Trimethylbenze	526-73-8	120.19	P	0.008	7%	0.996
82	1,2-Dichlorobenzene	95-50-1	147.00	T	0.007	5%	0.998
83	m-Diethylbenzene	141-93-5	134.22	P	0.006	6%	0.998
84	p-Diethylbenzene	105-05-5	134.22	P	0.006	5%	0.997
85	1,2,4-Trichlorobenzene	120-82-1	181.45	T	0.008	6%	0.996
86	Naphthalene	91-20-3	128.18	T	0.012	4%	0.998
**OVOCs**							
87	Acetone	67-64-1	58.08	T	0.009	3%	0.996
88	Isopropanol	67-63-0	60.10	T	0.002	6%	0.995
89	MTBE	1634-04-4	88.15	T	0.006	4%	0.993
90	Vinyl acetate	108-05-4	86.09	T	0.005	5%	0.993
91	2-Butanone	78-93-3	72.11	T	0.003	5%	0.992
92	Ethyl acetate	141-78-6	88.11	T	0.006	5%	0.995
93	1,4-Dioxane	123-91-1	88.11	T	0.004	5%	0.994
94	Methyl methacrylate	80-62-6	100.12	T	0.002	6%	0.998
95	Methyl isobutyl ketone	108-10-1	100.16	T	0.002	4%	0.997
96	2-Hexanone	591-78-6	100.16	T	0.003	6%	0.996
**Sulfide**							
97	Carbon disulfide	75-15-0	76.14	T	0.004	6%	0.995

[a] 2,3-dimethylbutane and 2-methylpentane, trans-2-pentene and cis-2-pentene, m-xylene and p-xylene co-eluted during the GC-MS analysis.

[b] **Molar.:** Molar mass, g/mol.

[c] **P or T:** P for PAMS standard substances[88], T for TO-15 standard substances[89].

Table S3. Calculation parameters related to the environment intergrated effects assessment of VOCs.

Compound	K_{OH}[26, 27]	MIR[28]	SOAP[27, 29, 30]	Toxicity[21, 90]
**Alkanes**				
iso-Butane	2.12	1.23	0.00	0.00
n-Butane	2.36	1.15	0.30	0.00
iso-Pentane	3.60	1.45	0.20	0.00
n-Pentane	3.80	1.31	0.30	0.00
2,2-Dimethylbutane	2.23	1.17	0.00	0.00
Cyclopentane	4.97	2.39	1.20	0.00
**2,3-Dimethylbutane**	5.78	0.97	0.00	0.00
**2-Methylpentane**	5.20	1.50	0.00	0.00
3-Methylpentane	5.20	1.80	0.20	0.00
n-Hexane	5.20	1.24	0.10	1.00
Methylcyclopentane	0.00	2.19	2.20	0.00
2,4-Dimethylpentane	4.77	1.55	0.00	0.00
Cyclohexane	6.97	1.25	2.20	0.00
2-Methylhexane	0.00	1.19	0.28	0.00
2,3-Dimethylpentane	4.77	1.34	0.40	0.00
3-Methylhexane	5.20	1.61	0.28	0.00
2,2,4-Trimethylpentane	3.34	1.26	0.73	1.00
n-Heptane	6.76	1.07	0.10	0.00
Methylcyclohexane	9.64	1.70	3.50	0.00
2,3,4-Trimethylpentane	6.60	1.03	0.73	0.00
2-Methylheptane	6.76	1.07	0.90	0.00
3-Methylheptane	6.76	1.24	0.90	0.00
n-Octane	8.11	0.90	0.80	0.00
Nonane	9.70	0.78	1.90	0.00
n-Decane	11.00	0.68	7.00	0.00
n-Undecane	12.30	0.61	16.20	0.00
n-Dodecane	13.20	0.55	34.50	0.00
**Alkenes**				
1-Butene	31.40	9.73	1.20	0.00
trans-2-Butene	64.00	15.16	4.00	0.00
cis-2-Butene	56.40	14.24	3.60	0.00
1-Pentene	31.40	7.21	2.60	0.00
Isoprene	100.00	10.61	1.90	1.00
**trans-2-Pentene**	67.00	10.56	3.10	0.00
**cis-2-Pentene**	65.00	10.38	3.10	0.00
1-Hexene	37.00	5.49	7.70	0.00
**Halocarbons**				
Methyl chloride	0.00	0.04	0.00	3.00
Dichlorotetrafluoroethane	0.00	0.00	0.00	0.00
Vinyl chloride	0.00	2.83	0.00	4.00
Bromomethane	0.00	0.02	0.00	1.00

Chloroethane	0.00	0.29	0.00	1.00
Trichlorofluoromethane	0.00	0.00	0.00	0.00
1,1-Dichloroethene	10.90	1.79	0.00	2.00
Dichloromethane	0.00	0.04	0.00	3.00
trans-1,2-Dichloroethylene	0.00	1.70	0.00	0.00
1,1-Dichloroethane	0.00	0.07	0.00	0.00
cis-1,2-Dichloroethylene	0.00	1.70	0.00	0.00
Trichloromethane	0.00	0.02	0.00	3.00
1,1,1-Trichloroethane	0.00	0.00	0.00	3.00
Tetrachloromethane	0.00	0.00	0.00	3.00
1,2-Dichloropropane	0.00	0.29	0.00	4.00
Bromodichloromethane	0.00	0.00	0.00	2.00
Trichloroethylene	2.36	0.64	0.00	4.00
cis-1,3-Dichloropropene	0.00	3.70	0.00	0.00
trans-1,3-Dichloropropene	0.00	5.03	0.00	0.00
1,1,2-Trichloroethane	0.00	0.09	0.00	1.00
Dibromochloromethane	0.00	0.00	0.00	1.00
1,2-Dibromoethane	0.00	0.10	0.00	3.00
Tetrachloroethene	0.00	0.03	0.00	2.00
Tribromomethane	0.00	0.00	0.00	1.00
1,1,2,2-Tetrachloroethane	0.00	0.00	0.00	2.00
Hexachloro-1,3-butadiene	0.00	0.00	0.00	1.00
**Aromatics**				
Tetrahydrofuran	0.00	4.31	0.00	2.00
Benzene	1.22	0.72	36.00	4.00
Toluene	5.63	4.00	100.00	2.00
Chlorobenzene	0.00	0.32	0.00	0.00
Ethylbenzene	7.00	3.04	111.60	2.00
**m-Xylene**	23.10	9.75	84.50	2.00
**p-Xylene**	14.30	5.84	84.50	2.00
Styrene	58.00	1.73	212.30	2.00
o-Xylene	13.60	7.64	95.50	2.00
Isopropylbenzene	6.30	2.52	95.50	1.00
n-Propylbenzene	5.80	2.03	109.70	1.00
m-Ethyltoluene	18.60	7.39	100.60	0.00
p-Ethyltoluene	11.80	4.44	69.70	0.00
1,3,5-Trimethylbenzene	56.70	11.76	13.50	1.00
o-Ethyltoluene	11.90	5.59	94.80	0.00
1,2,4-Trimethylbenzene	32.50	8.87	20.60	1.00
Benzyl chloride	0.00	2.92	0.00	3.00
1,3-Dichlorobenzene	0.00	0.18	0.00	1.00
1,4-Dichlorobenzene	0.00	0.18	0.00	2.00
1,2,3-Trimethylbenze	32.70	11.97	43.90	1.00
1,2-Dichlorobenzene	0.00	0.18	0.00	1.00

m-Diethylbenzene	0.00	7.10	10.00	0.00
p-Diethylbenzene	0.00	4.43	10.00	0.00
1,2,4-Trichlorobenzene	0.00	0.09	0.00	0.00
Naphthalene	23.00	3.34	36.00	2.00
**OVOCs**				
Acetone	0.17	0.36	0.30	0.00
Isopropanol	5.10	0.61	0.40	0.00
MTBE	2.94	0.73	0.00	2.00
Vinyl acetate	0.00	3.20	0.00	2.00
2-Butanone	1.22	1.48	0.60	1.00
Ethyl acetate	0.00	0.63	0.10	0.00
1,4-Dioxane	10.90	2.62	0.00	2.00
Methyl methacrylate	0.00	15.61	0.00	1.00
Methyl isobutyl ketone	13.00	3.88	0.60	2.00
2-Hexanone	9.10	3.14	0.00	0.00
**Sulfide**				
Carbon disulfide	0.00	0.25	0.00	0.00

Table S4. VOCs species included in PMF source apportionment modeling.

NO.	Compound	NO.	Compound
1	Acetone	46	cis-1,2-Dichloroethylene
2	Isopropanol	47	Trichloromethane
3	MTBE	48	1,1,1-Trichloroethane
4	Vinyl acetate	49	Tetrachloromethane
5	2-Butanone	50	1,2-Dichloropropane
6	Ethyl acetate	51	Trichloroethylene
7	1,4-Dioxane	52	cis-1,3-Dichloropropene
8	Methyl methacrylate	53	trans-1,3-Dichloropropene
9	Methyl isobutyl ketone	54	1,1,2-Trichloroethane
10	2-Hexanone	55	1,2-Dibromoethane
11	Tetrahydrofuran	56	Tetrachloroethene
12	Benzene	57	1,1,2,2-Tetrachloroethane
13	Toluene	58	iso-Butane
14	Chlorobenzene	59	n-Butane
15	Ethylbenzene	60	iso-Pentane
16	m/p-Xylene	61	n-Pentane
17	Styrene	62	2,2-Dimethylbutane
18	o-Xylene	63	Cyclopentane
19	Isopropylbenzene	64	2,3-Dimethylbutan
20	n-Propylbenzene	65	3-Methylpentane
21	m-Ethyltoluene	66	n-Hexane
22	p-Ethyltoluene	67	Methylcyclopentane
23	1,3,5-Trimethylbenzene	68	2,4-Dimethylpentane
24	o-Ethyltoluene	69	Cyclohexane

25	1,2,4-Trimethylbenzene	70	2-Methylhexane
26	Benzyl chloride	71	2,3-Dimethylpentane
27	1,3-Dichlorobenzene	72	3-Methylhexane
28	1,4-Dichlorobenzene	73	2,2,4-Trimethylpentane
29	1,2,3-Trimethylbenze	74	n-Heptane
30	1,2-Dichlorobenzene	75	Methylcyclohexane
31	m-Diethylbenzene	76	2,3,4-Trimethylpentane
32	p-Diethylbenzene	77	2-Methylheptane
33	1,2,4-Trichlorobenzene	78	3-Methylheptane
34	Naphthalene	79	n-Octane
35	Carbon disulfide	80	Nonane
36	Methyl chloride	81	n-Decane
37	Dichlorotetrafluoroethane	82	n-Undecane
38	Vinyl chloride	83	n-Dodecane
39	Bromomethane	84	1-Butene
40	Chloroethane	85	trans-2-Butene
41	Trichlorofluoromethane	86	cis-2-Butene
42	1,1-Dichloroethene	87	1-Pentene
43	Dichloromethane	88	Isoprene
44	trans-1,2-Dichloroethylene	89	trans/cis-2-Pentene
45	1,1-Dichloroethane	90	1-Hexene

Table S5. Summary of the PMF error estimation diagnostics using VOCs dataset in Harbin.

**Diagnostic**	**Non-heating**	**Heating**
Factor number	5	5
Q_{true}	10181.6	7303.17
Q_{robust}	9716.54	6765.38
$Q_{true}/Q_{expected}$	1.24	1.52
DISP %dQ	-0.049	-0.089
DISP swaps	0	0
Factors with the lowest BS mapping	97%	94%
Percentage of cases accepted in BS-DISP	89%	94%
# of swaps in BS-DISP	11	6

Table S6. Mapping[a] of BS factors to the base factors at a 5-factor solution for Non-heating dataset.

	**Vehicle exhaust**	**Combustion source**	**Solvent & coating**	**Solvent & fuel evaporation**	**Petrochemical industry source**
Vehicle exhaust	**98**	0	0	1	1
Combustion source	0	**98**	1	0	1
Solvent & coating	0	1	**97**	1	1
Solvent &fuel	0	0	0	**100**	0

evaporation					
Petrochemical industry source	0	0	0	1	**99**

[a] Minimum correlation R-value for bootstrap was 0.6.

Table S7. Mapping[a] of BS factors to the base factors at a 5-factor solution for Heating dataset.

	**Vehicle exhaust**	**Combustion source**	**Solvent & coating**	**Solvent & fuel evaporation**	**Petrochemical industry source**
Vehicle exhaust	**98**	0	1	1	0
Combustion source	1	**94**	1	0	4
Solvent & coating	0	1	**99**	0	0
Solvent &fuel evaporation	0	0	0	**100**	0
Petrochemical industry source	0	0	0	0	**100**

[a] Minimum correlation R-value for bootstrap was 0.6.

Table S8. The REL and CPF values of the selected hazardous VOCs and target organs.

**NO.**	**Compound**	**REL**[34]	**CPF**[34]	**Target organs**[34]
1	Isopropanol	7000		kidney; development
2	Methyl Tert-Butyl Ether	8000	0.0018	kidney; eyes; alimentary system (liver)
3	Vinyl Acetate	200		respiratory system
4	1,4-Dioxane	3000	0.027	alimentary system; kidney; cardiovascular system
5	Benzene	3	0.1	hematologic system
6	Toluene	420		eyes
7	Chlorobenzene	1000		alimentary system (liver); kidney; reproductive system
8	Ethylbenzene	2000	0.0087	alimentary system (liver); kidney; endocrine system; development
9	Benzyl Chloride		0.17	
10	m/p-Xylene	700		nervous & respiratory systems; eyes
11	Styrene	900		nervous system
12	o-Xylene	700		nervous & respiratory systems; eyes
13	1,3,5-Trimethylbenzene	4		nervous system
14	1,2,4-Trimethylbenzene	4		nervous system
15	1,4-Dichlorobenzene	800	0.04	nervous & respiratory; alimentary systems (liver); kidney
16	1,2,3-Trimethylbenze	4		nervous system
17	Naphthalene	9	0.12	respiratory system
18	Vinyl chloride		0.27	
19	Carbon disulfide	800		nervous system; reproductive system
20	Dichlorotetrafluoroethane	13		bone and teeth; respiratory system
21	Bromomethane	5		respiratory system; nervous system; development
22	Chloroethane	30000		bone and teeth

23	Trichlorofluoromethane	13		bone and teeth; respiratory system
24	1,1-Dichloroethene	70		alimentary system (liver)
25	Dichloromethane	400	0.0035	cardiovascular system; nervous system
26	1,1-Dichloroethane		0.0057	
27	Trichloromethane	300	0.019	alimentary system; kidney; development
28	1,1,1-Trichloroethane	1000		nervous system
29	Carbon tetrachloride	40	0.15	alimentary and nervous systems; development
30	Trichloroethylene	600	0.007	nervous system; eyes
31	1,1,2-Trichloroethane		0.057	
32	1,2-Dibromoethane	0.8	0.25	reproductive system
33	Perchloroethylene	35	0.021	kidney; alimentary system (liver)
34	1,1,2,2-Tetrachloroethane		0.2	
35	Isoprene		0.019	
36	n-Hexane	7000		nervous system

Table S9. Recommended parameter values for cancer risk calculation for different age groups.

**Parameter**[34]	**Unit**	**Age (years)**					
		**3rd Trimester**	**0-2**	**2-9**	**2-16**	**16-30**	**16-70**
BR/BW	L/kg-day	361	1090	861	745	335	290
A	unitless	1	1	1	1	1	1
EF	unitless	0.96	0.96	0.96	0.96	0.96	0.96
ASF	unitless	10	10	3	3	1	1
ED	year	0.25	2	7	14	14	54
AT	year	70	70	70	70	70	70
FAH	unitless	0.85	0.85	0.72	0.72	0.73	0.73
